# Acute Effects of Traditional Versus Cluster Set Upper Body Resistance Training on Heart Rate Variability and Blood Pressure in Trained Men

**DOI:** 10.1002/ejsc.70006

**Published:** 2025-06-26

**Authors:** Ali K. Güngör, Hüseyin Topçu, Andrew A. Flatt

**Affiliations:** ^1^ Faculty of Sport Science Bursa Uludag University Bursa Turkey; ^2^ Department of Health Sciences and Kinesiology Georgia Southern University Savannah Georgia USA

**Keywords:** autonomic nervous system, cardiovascular response, recovery, resistance exercise, set configuration

## Abstract

Traditional (TRD) and cluster set (CLT) resistance training (RT) configurations differentially affect cardiovascular parameters, such as heart rate variability (HRV) and blood pressure (BP), but the cardiovascular effects of upper body TRD and CLT with multiple exercises remain unclear. To compare the acute effects of upper body TRD and CLT on postexercise HRV and BP variables. Sixteen men with ≥ 1 year of RT experience participated in this randomized crossover study. Subjects performed four upper‐body exercises in both protocols, matched for volume, intensity, and rest periods. HRV and BP were measured pre‐exercise, postexercise, and again every 10 min for 40 min postexercise. Heart rate was elevated in both conditions until 30 min for TRD, but recovered by 20 min for CLT, and was lower in CLT versus TRD at 20–40 min (*p* values < 0.05). Root mean square of successive differences was reduced in both conditions until 30 min in TRD, but recovered by 20 min in CLT, with higher values in CLT versus TRD at 20–40 min (*p* values < 0.05). Despite no interaction (*p* > 0.05), systolic BP (SBP) was higher overall in CLT (*p* < 0.05). Moreover, effect sizes revealed *moderate* SBP reductions from pre‐exercise across all postexercise time points in TRD, with SBP lower in TRD versus CLT at 20–40 min (*small*‐to‐*moderate* effect sizes). CLT promoted faster cardiac‐autonomic recovery, whereas TRD tended to promote greater postexercise hypotension. Thus, set configuration should be selected based on specific goals, such as accelerating parasympathetic reactivation or reducing SBP.

## Summary

- Volume‐, intensity‐, and rest‐matched upper‐body resistance exercise performed in a traditional and cluster‐set format differently affected heart rate variability (HRV) and blood pressure (BP) in trained men.
- Reductions in vagal‐related HRV and BP were slower to recover to baseline following the traditional resistance exercise protocol.
- Despite an equal amount of work in similar time, cluster‐set training facilitates faster postexercise recovery of cardiac‐parasympathetic and hemodynamic parameters.

## 1 Introduction

Resistance training (RT) is recommended as a nonpharmacological approach for both the prevention and treatment of cardiovascular disorders as well as for enhancing athletic performance, improving physical fitness, and reducing the risk of injury (G. Haff and Triplett 2015). Moreover, chronic RT has been shown to improve various markers of cardiac autonomic control, inferred from heart rate variability (HRV), in clinical populations (Selig et al. 2004). HRV is a reliable noninvasive tool for evaluating cardiac autonomic modulation during and after exercise (Stanley et al. 2013) and is defined as the oscillation in the intervals between consecutive R‐waves (Malik et al. 1996). During exercise, sympathetic activity increases whereas parasympathetic activity decreases, causing a reduction in HRV. After exercise, there is a gradual reactivation of the parasympathetic system accompanied by sympathetic withdrawal. The return of HRV to baseline is thought to reflect the restoration of cardiovascular homeostasis, a key component of autonomic and global recovery (Stanley et al. 2013). Contrastingly, excessive or prolonged alterations in cardiac autonomic modulation can increase the risk of cardiac arrhythmias in the postexercise period (Von Klot et al. 2008). The autonomic nervous system also plays a crucial role in regulating and maintaining systolic (SBP) and diastolic blood pressure (DBP). During exercise, the sympathetic nervous system secretes norepinephrine, increasing heart rate, myocardial contractility, and BP. After acute RT, a decrease in BP is often observed (i.e., postexercise hypotension) (Rezk et al. 2006), which is therapeutic for patients with high blood pressure (Brito et al. 2018). However, acute BP and HRV responses to RT vary based on the number of repetitions, sets, and rest intervals between sets, intensity, and volume (Güngör et al. 2024; Rúa‐Alonso et al. 2020). Since cardiovascular adjustments after exercise have clinical implications (Brito et al. 2018; Von Klot et al. 2008), further study is needed to determine how various RT protocols affect HRV and BP.

A frequently overlooked and underutilized aspect of RT programs is the manipulation of set structure (G. G. Haff et al. 2008). For example, the number of repetitions, training load, and rest periods within a set can be adjusted to modify the training stimulus. Set structures are generally classified into two main categories: traditional sets (TRD) and cluster sets (CLT) (G. G. Haff et al. 2008). In TRD, all repetitions are performed consecutively, with rest provided only between sets. In contrast, CLT includes planned rest intervals within sets, in addition to the standard rest periods between sets (Tufano et al. 2017). Although performing TRD to or near muscle failure may provide a greater hypertrophic stimulus for skeletal muscle (Pareja‐Blanco et al. 2020), consecutive repetitions in TRD are associated with increased sympathetic activation and a simultaneous rise in arterial BP (Rúa‐Alonso et al. 2022), along with a gradual decline in mechanical performance (Gomides et al. 2010). Peak SBP during the first repetition increases as the workload progressively rises with successive repetitions (Sale et al. 1994), and set duration has been proposed as a major factor influencing SBP workload (Lovell et al. 2011). Consequently, reducing the number of repetitions per set at the same exercise intensity may reduce mechanical performance loss and limit sympathovagal imbalance (Iglesias‐Soler et al. 2015). Nevertheless, only a few studies have investigated the effects of TRD and CLT matched for volume, intensity, and rest periods on HRV and BP (Iglesias‐Soler et al. 2015; Mayo, Iglesias‐Soler, Carballeira‐Fernández, et al. 2016; Mayo, Iglesias‐Soler, Fariñas‐Rodríguez, et al. 2016; Rúa‐Alonso et al. 2020). One study found that SBP was lower for CLT compared to TRD during squat exercises, but no significant between‐group differences were observed in HRV (Iglesias‐Soler et al. 2015). Another study using a crossover design reported delayed HRV recovery after bench pressing for TRD (failure set) compared to CLT and a control session, whereas SBP and DBP were lower for TRD versus control (Mayo, Iglesias‐Soler, Fariñas‐Rodríguez, et al. 2016). Meanwhile, another study showed that despite more suppressed HRV parameters after TRD versus CLT, similar BP responses were observed (Rúa‐Alonso et al. 2022). Thus, recent findings are somewhat conflicting regarding the postexercise cardiovascular effects of CLT versus TRD, particularly for BP parameters, warranting further investigation.

Previous studies have examined the effects of variable set configurations on HRV and BP parameters where volume, intensity, and rest periods were equalized, typically using single (Iglesias‐Soler et al. 2015; Mayo, Iglesias‐Soler, Carballeira‐Fernández, et al. 2016; Mayo, Iglesias‐Soler, Fariñas‐Rodríguez, et al. 2016) or whole‐body exercises (Rúa‐Alonso et al. 2020). To the best of our knowledge, there is no study examining the effect of volume‐, intensity‐, and total rest interval‐matched TRD and CLT consisting of multiple exercises targeting the upper body. This research is needed because many individuals perform split routines in which upper and lower body RT is performed on separate days, indicating that half of their sessions could be upper body only. Notably, the smaller vascular networks in the upper body may create greater resistance to blood flow, potentially overloading the cardiovascular system during upper‐body exercises (Toner et al. 1990). Moreover, high volume upper body RT has been shown to negatively affect some autonomic and vascular markers relative to lower body training (Li et al. 2015; Okamoto et al. 2009). Determining the effects of different upper body protocols with matched volume, intensity, and total rest periods on cardiovascular variables may provide useful information for designing and prescribing RT programs. Therefore, the purpose of this study was to compare the effects of TRD and CLT, performed with equal volume, intensity, and rest periods, on postexercise HRV and BP parameters in trained men. We hypothesized that CLT would cause smaller alteration in HRV and BP compared to TRD.

## 2 Material and Methods

### 2.1 Study Design

Subjects visited the laboratory on four separate days, with at least 72 h between each visit. All sessions were performed in the Athletic Performance Laboratory at Bursa Uludağ University (temperature 22°C–24°C and humidity 33%–45%) between 10:00 a.m. and 12:00 p.m. to control for time of day. Subjects' 6 repetition maximum (RM) and 6RM retest measurements were recorded during the first two visits. On the third and fourth visits, subjects completed the TRD and CLT protocols in a counterbalanced cross‐over design. A computer‐generated random number sequence was used to randomize the first 8 participants to TRD or CLT, with the subsequent 8 participants performing the opposite order so that half of the group performed TRD first and vice versa. The research team was aware of each subjects' assigned sequence. The exercises performed included the barbell bench press (BnP), dumbbell fly (DF), incline barbell bench press (IBnP), and decline dumbbell bench press (DBnP), utilizing both TRD and CLT methods. HRV was assessed at pre‐interval, immediately post‐interval (3 min), and at 10 min interval for a total duration of 40 min following the exercise bout. The BP parameters (SBP, DBP, and mean arterial pressure (MAP)) were measured immediately following HRV recordings at the same time points. Subjects were instructed to abstain from caffeine, alcohol, and intense exercise for at least 24 h prior to the exercise sessions, refrain from food consumption for 3 h, and liquid intake for 1 h before the exercise (Christiani et al. 2021).

### 2.2 Subjects

Young adult men with at least 12 months of RT experience were recruited from local training facilities for the study. Inclusion criteria for the subjects were as follows: (a) no joint or bone injuries within the last 6 months, (b) no cardiovascular diseases or use of medications or substances affecting the cardiovascular system, (c) no consumption of stimulants (e.g., caffeine) or creatine, and (d) no metabolic diseases (Chobanian et al. 2003). Subjects with resting SBP ≥ 140 mmHg or diastolic blood pressure (DBP) ≥ 90 mmHg were excluded (Whelton et al. 2018). Body weight (kg), body mass index (BMI, kg/m^2^), and body fat percentage were assessed using a body composition analyzer (Tanita Model BF‐350; Tanita Corp., Tokyo, Japan). For the analysis, subjects' clothing weight, gender, age, and height were entered into TANITA, after which the subjects stepped on the foot sensors and held the handles. Measurements were recorded using “athletic mode”. Before the exercise sessions, subjects received a detailed explanation of the study procedures, requirements, potential benefits, and risks prior to signing an informed consent form. The study followed the principles outlined in the Declaration of Helsinki and was approved by the local Clinical Research Ethics Committee (Approval code: 2021‐18/14), adhering to the ethical standards of the World Medical Association. A priori power analysis was conducted using the G Power software (version 3.1.9.7) for the *F*‐test family (repeated measures ANOVA, within‐subject factors) to determine the required sample size. We specifically referenced Holmes et al. (2022) which used a sample of 10 participants and reported a medium effect size. Based on this, we selected an effect size of *f* = 0.30. The power analysis indicated that a sample size of 14 participants across two sessions with six measures would achieve a statistical power of *β* = 0.80 at an alpha level of *α* = 0.05. To account for potential participant dropouts, we recruited 16 participants, which provided sufficient power (> 80%) to detect the specified effect size (Beck 2013).

### 2.3 Procedures

#### 2.3.1 6RM Test

A test protocol was implemented to determine subjects 6RM in the BnP, DF, IBnP, and DBnP exercises. Exercise technique was carried out as previously described (Hedrick 2019; Tumminello 2022). Testing loads were based of subjects self‐estimated 6RM load for each exercise and were subsequently adjusted according to performance. Before 6RM testing, subjects underwent a 5 min warm‐up routine to reduce the risk of injury and increase muscle activation. The warm‐up included 2 min of light jogging, 15 repetitions at 40% of their estimated 1RM, and eight repetitions at 70% of their 1RM, with 1 min of active rest in between. During each exercise, subjects attempted to lift their estimated 6RM load. If a subject was able to complete more than six repetitions, the weight was increased by 5%–10%; if they were unable to complete six repetitions, the weight was decreased by 5%–10%, and the test was re‐executed. After each trial, subjects were given a 5 min rest period to recover. The highest load that subjects successfully lifted for six repetitions was recorded as the final 6RM.

#### 2.3.2 Training Interventions

**FIGURE 1:**
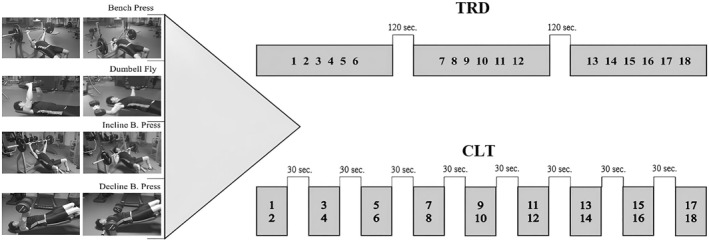
TRD and CLT practice protocols.

Experimental RT sessions occurred 72 h after the 6RM test. Sessions began with the same warm‐up procedure that preceded 6RM testing. After the warm‐up routine, a 120 s rest period was allowed before beginning the RT intervention. In the TRD protocol, each exercise was performed with 3 sets of 6 repetitions at 6RM, 120 s rest between sets, and 5 min rest between exercises. In the CLT protocol, each exercise was performed with 9 sets of 2 repetitions at 6RM, 30 s rest between sets, and 5 min rest between exercises. Each repetition included a 2 s eccentric phase followed by a concentric action performed as quickly as possible to maximize the intended speed. Each TRD set was completed near or to muscular failure, defined as the mechanical inability to complete a repetition and the subject choosing to stop due to the perception that they could not continue the exercise (Refalo et al. 2022). During the exercises, subjects received verbal encouragement and minimal assistance was provided by a researcher when necessary. In each exercise session, subjects performed BnP, DF, IBnP (bench angle: +30°), and DBnP (bench angle: −15°) exercises, respectively. In addition, volume, intensity, and rest periods were equalized in both RT sessions. RT protocols are shown in Figure 1. HRV and BP were measured before and after exercise as described below.

#### 2.3.3 Heart Rate Variability Assessment

The measurement of HR through successive R–R intervals was recorded using a HR monitor (Polar V800 with an H10 strap, Polar Electro OY, Kempele, Finland) (Giles et al. 2016). Average and peak HR values during the RT protocols were recorded to index internal load. Time points for HRV data collection were as follows: pre: 10 min (5–10 min after a 5 min stabilization) and post: 3 min (one to three min), 10 min (5–10 min), 20 min (15–20 min), 30 min (25–30 min), and 40 min (35–40 min) (Esco and Flatt 2014). Measurements were performed in the supine position whereas subjects remained quiet, still, and breathed naturally. R–R interval data were subsequently transferred to a computer via the Polar Flow application for analysis. Kubios HRV software (Standard version 3.5.0, Biosignal Analysis and Medical Imaging Group, Department of Physics, University of Kuopio, Kuopio, Finland) was used to calculate HRV parameters. The software automatically performed smoothness priors detrending procedures (Tarvainen et al. 2002), removed noise, and applied artifact correction with a very low correction threshold (not exceeding 2% in the current sample). To estimate cardiac autonomic modulation, the following parameters were recorded: mean‐HR, which indicates the average heart rate during the measurement period; root‐mean square of successive differences (RMSSD), which predominantly provides information about the parasympathetic system; and standard deviation of normal R–R intervals (SDNN), which reflects overall heart rate variability and represents both sympathetic and parasympathetic influences (Malik et al. 1996).

#### 2.3.4 Blood Pressure Assessment

Supine brachial SBP, DBP, and MAP were measured using an automated oscillometric device (Omron M2 HEM‐7121‐E, Kyoto, Japan). The equipment was automatically calibrated before each use. Measurements were taken from the left arm in accordance with the guidelines provided by the American Heart Association (Pickering et al. 2005). BP measurements were performed immediately following the HRV assessment at the same pre‐exercise and postexercise time points.

#### 2.3.5 Statistical Analysis

Data analysis was conducted using the SPSS version 28.0 software (IBM Corp., Armonk, NY, USA). Descriptive parameters were presented as means and standard deviations. The Shapiro–Wilk test was employed to assess the normal distribution of the parameters. To determine the reliability of the 6RM test, intraclass correlation coefficients (ICCs, [3,1] and two‐way mixed‐effects model, consistency, single measures) with 95% confidence intervals were calculated for the BnP, DF, IBnP, and DBnP. ICCs were classified as follows: poor reliability (ICC ≤ 0.50), moderate reliability (0.50 < ICC ≤ 0.75), good reliability (0.75 < ICC ≤ 0.90), and excellent reliability (ICC > 0.90) (Koo and Li 2016). Training duration and internal load were compared between TRD and CLT with paired *t*‐tests. A two‐way repeated measures ANOVA (2 sessions × 6 times) was used to evaluate the HRV and BP parameters. When appropriate, post hoc multiple comparisons were conducted with Bonferroni adjustments and the significance level was set at *p* < 0.05. Effect size (ES) was calculated using partial eta‐squared ($n_{p}^{2}$) from the repeated measures ANOVA. Standardized differences were also calculated for pairwise comparisons using Hedges' g effect sizes, which were interpreted based on thresholds outlined by Hopkins et al. (2009): values ≤ 0.19 were considered trivial,  ≤ 0.59 as small,  ≤ 1.19 as moderate,  ≤ 1.99 as large, and ≥ 2.0 as very large.

## 3 Results

**FIGURE 2:**
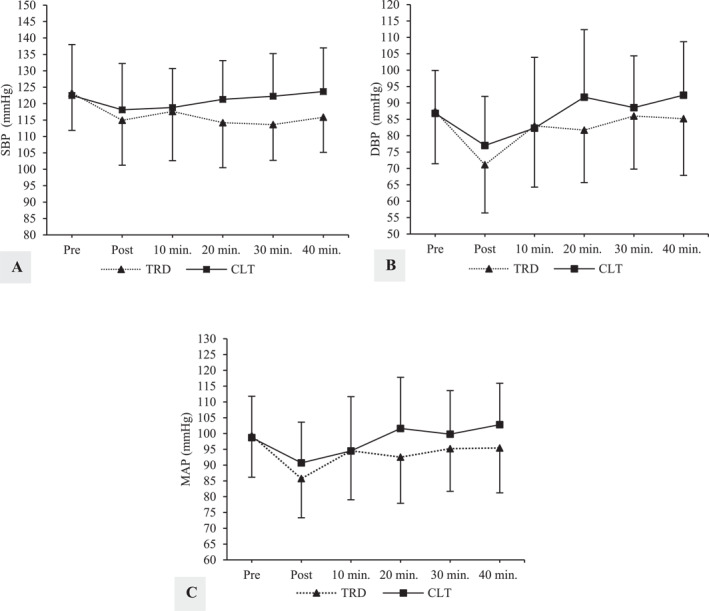
Mean ± standard deviation for hemodynamic parameters across time points for traditional and cluster set protocols in young resistance‐trained men (A: SBP, B: DBP, and C: MAP). TRD; traditional set, CLT; cluster set, SBP; systolic blood pressure, DBP; diastolic blood pressure, and MAP; mean arterial pressure.

Descriptive statistics of the subjects are shown in Table 1. The training experience of the subjects ranged from 1 to 4 years (3–4 days per week training frequency). Although no subjects reported being diagnosed with hypertension, 7/16 subjects exhibited pre‐exercise SBP values that were elevated and 15/16 subjects exhibited pre‐exercise DBP values that meet stage 1 hypertension categorization (Whelton et al. 2018) (Figure 2).

**TABLE 1:** Descriptive characteristics and 6RM values of the subjects.

Variables	Mean ± SD	Range
Age (year)	21.5 ± 2.2	18–28
Height (cm)	176 ± 8.1	158–187
Weight (kg)	76.5 ± 10.7	58.3–99.9
BMI (kg·m^2^)	24.5 ± 2.8	21.1–30.2
Body fat (%)	10.2 ± 4.2	3.3–18.2
Training experience (year)	2.3 ± 1.1	1–4
6RM BnP (kg)	80.9 ± 12.8	60–100
6RM DF (kg)	52.5 ± 4.1	45–60
6RM IBnP (kg)	68.1 ± 9.8	52–82
6RM DBnP (kg)	64.1 ± 4.9	60–72

Abbreviations: BMI = body mass index; BnP = bench press; DBnP = decline bench press; DF = dumbbell fly; IBnP = incline bench press; RM = repetition maximum; SD = standard deviation.

The 6RM test and retest ICC was BnP = 0.96, DF = 0.94, IBnP = 0.94, and DBnP = 0.98.

### 3.1 Training Duration

Total training duration was significantly shorter (*p* = 0.001 and *d* = 3.66) for TRD (35:03 ± 0:44 min:sec) versus CLT (36:58 ± 0:41 min:sec), although this difference was < 2 min.

### 3.2 Internal Load

Mean exercising HR was not different (*p* = 0.819, *g* = 0.08, and 95% CI [‐0.48, 0.64]) between TRD (122.4 ± 14.2 beats∙min^−1^) and CLT (121.2 ± 15.2 beats∙min^−1^). Similarly, peak exercising HR was not different (*p* = 0.077) between TRD (196.5 ± 11.4 beats∙min^−1^) and CLT (188.0 ± 20.0 beats∙min^−1^), although a small standardized difference was noted (*g* = 0.51 and 95% CI [‐0.05, 1.07]).

### 3.3 HRV Responses

Significant condition × time interactions were observed for Mean‐HR and RMSSD (Table 2). Post hoc analyses for Mean‐HR showed that relative to pre‐exercise, values were elevated until 30 min postexercise for TRD (*p*_post_ < 0.001, *g* = 3.64, and 95% CI [2.66, 4.63]; *p*_10min_ = 0.002, *g* = 1.04, and 95% CI [0.43, 1.65]; and *p*_20min_ = 0.004, *g* = 0.88, and 95% CI [0.28, 1.49]), whereas values were elevated until only 20 min postexercise for CLT (*p*_post_ < 0.001, *g* = 3.17, and 95% CI [2.23, 4.12] and *p*_10min_ = 0.002, *g* = 0.99, and 95% CI [0.39, 1.60]). Furthermore, Mean‐HR was significantly lower for CLT versus TRD at 20 , 30 , and 40 min postexercise (*p*_20min_ = 0.035, *g* = 0.38, and 95% CI [‐0.20, 0.96]; and *p*_30min_ = 0.023, *g* = 0.35, and 95% CI [‐0.23, 0.93]; and *p*_40min_ = 0.006, *g* = 0.58, and 95% CI [‐0.01, 1.16]). Post hoc analyses for RMSSD showed that relative to pre‐exercise, values were suppressed until 30 min post exercise for TRD (*p*_post_ < 0.001, *g* = 2.37, and 95% CI [1.59, 3.14]; *p*_10min_ = 0.005, *g* = 1.71, and 95% CI [1.04, 2.38]; and *p*_20min_ = 0.006, *g* = 1.52, and 95% CI [0.87, 2.18]), whereas values were suppressed until only 20 min post exercise for CLT (*p*_post_ < 0.001, *g* = 1.79, and 95% CI [1.11, 2.46] and *p*_10min_ = 0.018, *g* = 0.80, and 95% CI [0.21, 1.40]). Moreover, RMSSD was significantly higher for CLT versus TRD at 20 , 30 , and 40 min postexercise (*p*_20min_ = 0.046, *g* = 0.58, and 95% CI [‐0.00, 1.16]; and *p*_30min_ = 0.016, *g* = 0.70, and 95% CI [0.11, 1.30]; and *p*_40min_ = 0.017, *g* = 0.62, and 95% CI [0.03, 1.22]).

**TABLE 2:** Comparison of cardiac autonomic and hemodynamic parameters by condition and time point in young resistance‐trained men.

Variables	Exercise	Pre	Post	10 min.	20 min.	30 min.	40 min.	Time (T) main effect	Condition (C) main effect	T × C Interactions
		Mean ± SD	Mean ± SD	Mean ± SD	Mean ± SD	Mean ± SD	Mean ± SD	*(F/* $n_{p}^{2}$ */P)*	*(F/* $n_{p}^{2}$ */P)*	*(F/* $n_{p}^{2}$ */P)*
Mean‐HR (bpm)	TRD	73.3 ± 8.3	121.7 ± 16.6	85.9 ± 14.8	82.7 ± 12.5	79.2 ± 10.3	77.1 ± 9.4	174.815 0.921 **<** **0.001** **^a^**	2.129 0.123 0.167	2.369 0.136 **0.047** **^a^**
	CLT	73.7 ± 7.1	120.4 ± 19.4	83.6 ± 12.1	78.5 ± 9.4	75.9 ± 8.6	72.5 ± 5.7			
RMSSD (ms)	TRD	45 ± 16	15 ± 7.4	21.5 ± 10.5	25.4 ± 8.3	30 ± 9.7	38 ± 13.9	24.365 0.619 **<** **0.001** **^a^**	3.736 0.199 0.072	3.560 0.192 **0.006** **^a^**
	CLT	47.6 ± 22.5	16.9 ± 8.1	29.2 ± 22.8	36.8 ± 26	41.7 ± 21.3	50.2 ± 23.8			
SDNN (ms)	TRD	51.5 ± 18.6	25.6 ± 12	29.3 ± 9.7	35.3 ± 8.7	38.9 ± 8.8	47.8 ± 13.2	26.928 0.642 **<** **0.001** **^a^**	2.673 0.151 0.123	1.585 0.096 0.175
	CLT	52.1 ± 15.1	30.9 ± 16.3	33.7 ± 16.8	42.3 ± 18.9	48 ± 16.9	54.3 ± 15			
SBP (mmHg)	TRD	123.3 ± 11.4	114.9 ± 13.7	117.6 ± 15	114.2 ± 13.7	113.6 ± 10.9	115.9 ± 10.7	1.455 0.088 0.215	6.858 0.314 **0.019** **^a^**	1.240 0.076 0.299
	CLT	122.5 ± 15.5	118.1 ± 14.1	118.8 ± 11.9	121.3 ± 11.8	122.3 ± 13	123.7 ± 13.3			
DBP (mmHg)	TRD	87.4 ± 16	71.1 ± 14.7	83 ± 18.7	81.7 ± 16	86 ± 16.2	85.2 ± 17.3	5.162 0.256 **<** **0.001** **^a^**	2.162 0.126 0.162	1.315 0.081 0.267
	CLT	86.8 ± 13.1	77 ± 15	82.3 ± 21.6	91.8 ± 20.6	88.6 ± 15.8	92.4 ± 16.3			
MAP (mmHg)	TRD	99.4 ± 13.2	85.7 ± 12.4	94.5 ± 15.5	92.5 ± 14.6	95.2 ± 13.5	95.4 ± 14.2	4.596 0.235 **<** **0.001** **^a^**	3.867 0.205 0.068	1.479 0.090 0.207
	CLT	98.7 ± 13.1	90.7 ± 12.9	94.5 ± 17.2	101.6 ± 16.2	99.8 ± 13.8	102.8 ± 13.1			

Abbreviations: DBP; diastolic blood pressure, MAP, mean arterial pressure, mean; arithmetic mean, RMSSD; the root of the mean of the square of the difference of the RR intervals, SBP; systolic blood pressure, SD; standard deviation, SDNN; standard deviation of NN intervals.

^a^ Significant difference (*p* < 0.05).

**FIGURE 3:**
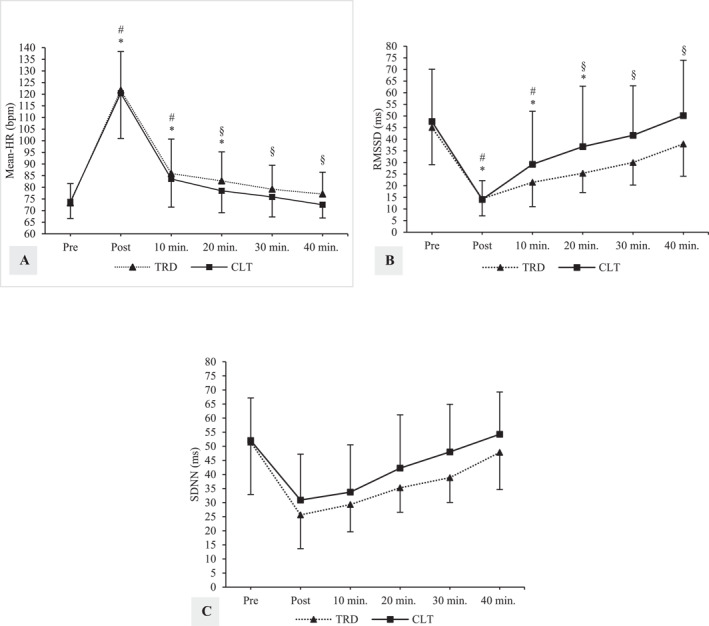
Mean ± standard deviation for cardiac autonomic parameters across time points for traditional and cluster set protocols in young resistance‐trained men (A: Mean‐HR, B: RMSSD, and C: SDNN). *Different from pre‐exercise values for TRD, ^#^different from pre‐exercise values for CLT, ^§^different between TRD and CLT, TRD; traditional set, CLT; cluster set, RMSSD; the root of the mean of the square of the difference of the RR intervals, and SDNN; standard deviation of normal R–R intervals.

Significant main effects of time were observed for Mean‐HR, RMSSD, and SDNN. Post hoc analyses for HR showed that relative to pre‐exercise, Mean‐HR was significantly increased at postexercise (*p* < 0.001, *g* = 3.55, and 95% CI [2.58, 4.53]), 10 min postexercise (*p* < 0.001, *g* = 1.08, and 95% CI [0.47, 1.69]), and 20min postexercise (*p* = 0.010, *g* = 0.79, and 95% CI [0.19, 1.39]). RMSSD showed significant reductions over time, with values significantly lower than pre‐exercise at postexercise (*p* < 0.001, *g* = 2.43, and 95% CI [1.64, 3.22]), 10 min postexercise (*p* = 0.006, *g* = 1.34, and 95% CI [0.66, 2.02]), and 20 min postexercise (*p* = 0.035, *g* = 0.92, and 95% CI [0.11, 1.73]). Similarly, SDNN showed significant reductions over time, with values significantly lower than pre‐exercise at postexercise (*p* < 0.001, *g* = 1.73, and 95% CI [1.00, 2.46]), 10 min postexercise (*p* = 0.005, *g* = 1.50, and 95% CI [0.76, 2.23]), and 20 min postexercise (*p* = 0.040, *g* = 0.94, and 95% CI [0.12, 1.75]). Although no significant interaction effect was observed for SDNN, Hedges' *g* analysis showed small to moderate reductions for TRD compared to CLT at all postexercise time points (*g*_post_ = 0.37 and 95% CI [‐0.21, 0.95]; *g*_10min_ = 0.32 and 95% CI [‐0.26, 0.90]; *g*_20min_ = 0.47 and 95% CI [‐0.11, 1.05]; *g*_30min_ = 0.67 and 95% CI [0.08, 1.27]; and *g*_40min_ = 0.45 and 95% CI [‐0.13, 1.03]). HRV values are displayed in Figure 3.

### 3.4 BP Responses

A significant main effect of condition was observed for SBP. Post hoc analysis showed that SBP was significantly higher for CLT compared to TRD (*p* = 0.019, *g* = 0.49, and 95% CI [‐0.09, 1.07]). Significant main effects of time were observed for DBP and MAP. Post hoc analysis showed that each were significantly reduced from pre‐exercise to postexercise (*p* = 0.042, *g* = 1.02, and 95% CI [0.42, 1.63] and *p* = 0.039, *g* = 0.96, and 95% CI [0.36, 1.56]). No significant condition × time interactions were observed for any BP parameters. Nevertheless, Hedges' g analysis showed trivial reductions in SBP relative to pre‐exercise for CLT at 20–40 min postexercise (*g*_20min_ = 0.09 and 95% CI [‐0.48, 0.65]; *g*_30min_ = 0.02 and 95% CI [‐0.54, 0.58]; and *g*_40min_ = 0.08 and 95% CI [‐0.48, 0.64]), whereas moderate decreases relative to pre‐exercise were observed at 20–40 min postexercise for TRD (*g*_20min_ = 0.71 and 95% CI [0.11, 1.31]; *g*_30min_ = 0.85 and 95% CI [0.24, 1.45]; and *g*_40min_ = 0.66 and 95% CI [0.06, 1.26]). Moreover, SBP for TRD was moderately lower than CLT at 20–40 min postexercise (*g*_20min_ = 0.55 and 95% CI [‐0.03, 1.13]; *g*_30min_ = 0.71 and 95% CI [0.11, 1.31]; and *g*_40min_ = 0.64 and 95% CI [0.05, 1.24]). All condition × time effect sizes for DBP and MAP ranged from trivial to small. BP values are displayed in Figure 2.

## 4 Discussion

The purpose of this study was to compare the acute effects of high‐volume upper body RT performed in a traditional or cluster set manner on postexercise HRV and BP parameters in trained individuals. Our main finding was that postexercise Mean‐HR and RMSSD recovery significantly varied as a function of exercise set configuration. Specifically, Mean‐HR and RMSSD were lower and higher, respectively, at 20 , 30 , and 40 min postexercise for CLT versus TRD. Although no significant interaction effects were observed for BP parameters, SBP was significantly lower overall for the CLT condition. Moreover, Hedges' effect size analysis revealed that moderate SBP reductions relative to pre‐exercise were observed at all postexercise time points for TRD, whereas trivial reductions were observed for CLT. These results tend to support our hypothesis that CLT would cause smaller alterations in HRV and BP relative to TRD.

Although HRV parameters were similarly altered for each condition immediately postexercise, CLT facilitated significantly faster HRV recovery to pre‐exercise values compared with TRD. Additionally, RMSSD exhibited a greater and more prolonged reduction than SDNN at post‐RT time points, especially in TRD, indicating that RT‐induced changes in HRV may be driven primarily by altered parasympathetic activity. Our findings add to a growing body of research showing that CLT is an effective strategy for minimizing the duration of RT‐induced disturbances in cardiac‐autonomic activity, without affecting total volume or intensity. Our results demonstrate that this observation also applies to RT sessions that involve a high volume of work distributed among multiple upper‐body exercises. In comparison to our study, Rúa‐Alonso et al. (2020) investigated the effects of TRD (4x10 repetitions, 120 s rest) and CLT (5x8 repetitions, 52 s rest) protocols involving five whole‐body exercises on HRV and BP parameters in 32 subjects with at least six months of RT experience. Similar to our findings, HRV parameters (RMSSD and SDNN) were significantly higher (*p* values < 0.05) for up to 40 min postexercise following the CLT compared to the TRD. In a study by Mayo, Iglesias‐Soler, Carballeira‐Fernández, et al. 2016, 17 subjects performed the leg press exercise using TRD (5x8 repetitions and 180 s rest), cluster set‐1 (CLT‐1, 10x4 repetitions, and 80 s rest), cluster set‐2 (CLT‐2, 40x1 repetition, and 18.5 s rest), and a control condition in a cross‐over design. In agreement with our findings, the natural log of RMSSD was significantly reduced until 30 min postexercise for CLT‐1 and through all 40 min postexercise time points for TRD compared to CLT‐2 and control (*p* values < 0.05). Given that our CLT configuration was most similar to CLT‐2, it seems that fewer repetitions per cluster is a key factor that facilitates accelerated postexercise cardiac‐autonomic recovery. Our findings are in partial agreement with another study showing that postexercise HRV was significantly higher for the CLT protocol compared to TRD following bench pressing (*p* = 0.027), whereas HRV was nonsignificantly higher (*p* > 0.05) for CLT versus TRD following the squat exercise (Mayo, Iglesias‐Soler, Fariñas‐Rodríguez, et al. 2016). In another study conducted with 10 judo athletes, RMSSD showed similar responses between CLT and TRD protocols; however, the HRV assessment was limited to one follow‐up time point at 8 min postexercise, which does not capture the recovery response that may occur at subsequent time points (Iglesias‐Soler et al. 2015).

Continuous (i.e., TRD) versus discontinuous repetitions (i.e., CLT) is the primary programming variable that appears to be driving the differences in post‐RT cardiac‐autonomic recovery given that total volume, intensity, rest periods, and internal load were matched between protocols. Although time duration was significantly longer for CLT, the difference was < 2 min, which has little practical relevance. The higher number of consecutive repetitions, and thus greater time under tension per set, in TRD likely led to an increased accumulation of glycolytic end‐products, such as lactate, which can influence autonomic responses. Based on the prescribed tempo (2 s eccentric phase and explosive concentric phase) time under tension per set would be ∼6 s for CLT and ∼18 s for TRD. Thus, CLT would be more phosphagen system‐dependent, whereas TRD would be more glycolytic system‐dependent (Baker et al. 2010). In support of this assertion, one study reported that lactate accumulation was ∼20% higher (*p* < 0.001) after TRD compared to CLT (Rúa‐Alonso et al. 2020). Moreover, a negative linear association (*r* = −0.64) has been observed between RMSSD and blood lactate concentration at 10 min postexercise in nine trained subjects following varying intensity RT protocols with or without vascular occlusion (Okuno et al. 2014). Increased concentrations of lactate and other metabolites stimulate groups III and IV nerve afferents in the exercising muscles, which triggers the metaboreflex and leads to an increase in sympathetic activity and a concurrent withdrawal of parasympathetic activity (Stanley et al. 2013). Thus, the physiological effects of higher anaerobic glycolytic processes on cardiac‐autonomic regulation could help explain why HRV parameters were slower to recover following TRD in our investigation.

In the present study, no significant condition × time interactions were observed for BP parameters. However, SBP was significantly higher overall in CLT versus TRD (condition effect), and DBP and MAP were significantly reduced immediately postexercise versus pre‐exercise (time effect). Moreover, Hedges' effect size analyses indicated that TRD promoted moderate SBP reductions from pre‐exercise through all postexercise time points, and that SBP was lower (effect size magnitudes ranging from small to moderate) for TRD versus CLT at 20–40‐min postexercise. Previous studies examining BP responses after TRD and CLT have reported inconsistent results. For instance, two studies showed no between‐condition differences in postexercise BP responses (Mayo, Iglesias‐Soler, Carballeira‐Fernández, et al. 2016; Rúa‐Alonso et al. 2020) whereas one study reported interaction effects. Specifically, Mayo, Iglesias‐Soler, Fariñas‐Rodríguez, et al. (2016) reported that SBP and DBP were significantly lower following a muscle failure protocol versus CLT at 30 and 35min postexercise, respectively. Discrepant findings may reflect variations in subject characteristics (e.g., health status such as normotensive vs. hypertensive), type of exercise performed (e.g., single vs. multiple exercises, or targeting upper, lower, or whole body), resistance training experience, age, and gender. In addition, postexercise hypotension following RT is thought to be influenced by factors, such as total exercise volume, muscle mass involved, and proximity to muscle failure, with training volume considered the primary factor (Figueiredo et al. 2015; Polito and Farinatti 2009). For example, De Souza et al. (2013) observed SBP, DBP, and MAP reductions only following a muscle failure protocol (volume was not equalized), suggesting that the decrease in BP was mainly due to higher training volumes. However, volume differences cannot explain the SBP effects observed for TRD in the current study. Our TRD protocol had overall significantly lower SBP and moderate postexercise effect size reductions in SBP through the 40 min period. We speculate that this may be due to greater metabolic stress and transient muscular occlusion from a longer time under tension per set during TRD. These effects promote reactive hyperemia and flow‐mediated vasodilation as a result of shear stress and nitric oxide signaling in the active tissue, thereby promoting acute reductions in SBP (Collier et al. 2010). Furthermore, unloading of arterial baroreceptors resulting from postexercise hypotension can evoke a reflex increase in heart rate and reduction in HRV through vagal withdrawal in an effort to maintain BP (Farinatti et al. 2021). This may help further explain our finding of a more prolonged suppression of parasympathetic HRV parameters following TRD versus CLT.

This study has some limitations. First, blood lactate concentration and perceived exertion were not measured. Second, HRV parameters were assessed in the supine position, both at rest and postexercise, yet different body positions may affect HRV and BP variables (de Tarso Veras Farinatti et al. 2009; Rabbani et al. 2021). Third, respiratory rate and tidal volume were not controlled, which can also affect HRV. Fourth, use of spotters during TRD may have had a small effect on mechanical load differences between groups. Lastly, only young trained men were included in this study, limiting the generalizability of our findings to this population. Future research should investigate the influence of blood lactate accumulation and other metabolic markers on postexercise HRV and BP responses. Moreover, future studies should examine cardiovascular responses in diverse populations with varying health conditions, training experience, and age ranges. Additionally, exploring other muscle groups and exercise intensities within these protocols could provide further insight into the optimal configuration for cardiovascular recovery following RT.

## 5 Conclusion

Our findings provide novel insight into the acute cardiovascular effects of upper body RT. The CLT protocol was associated with accelerated cardiac‐autonomic recovery, inferred from significantly higher postexercise RMSSD and lower Mean‐HR compared to TRD. Moreover, a tendency for greater post‐RT systolic hypotension was observed following TRD compared to CLT based on effect size analysis. Differences in HRV and BP responses were observed despite matched volume, intensity, and total rest periods between protocols. From a clinical perspective, our findings may support the use of CLT‐style resistance training for patients who are at a risk of dysrhythmia during prolonged postexercise parasympathetic withdrawal. Moreover, postexercise hypotension may (e.g., for hypertensive individuals without cardiac complications) or may not (e.g., for individuals with postural orthostatic hypotension) be desirable for different individuals. Thus, our findings may help guide RT programming considerations for targeting the desired cardiac‐autonomic and hemodynamic response for a specific population.

## Ethics Statement

This study was approved by Bursa Uludag University Faculty of Medicine Clinical Research Ethics Committee (Ethics Approval No: 2021‐18/14).

## Conflicts of Interest

The authors declare no conflicts of interest.

## Data Availability

The data will be made available by the author upon reasonable request via email.

## References

[ejsc70006-bib-0001] Baker, J. S. , M. C. McCormick , and R. A. Robergs . 2010. “Interaction Among Skeletal Muscle Metabolic Energy Systems During Intense Exercise.” Journal of Nutrition and Metabolism 2010, no. 1: 1–13. 10.1155/2010/905612. PMC3005844 21188163

[ejsc70006-bib-0002] Beck, T. W . 2013. “The Importance of A Priori Sample Size Estimation in Strength and Conditioning Research.” Journal of Strength & Conditioning Research 27, no. 8: 2323–2337. 10.1519/JSC.0B013E318278EEA0. 23880657

[ejsc70006-bib-0003] Brito, L. C. , R. Y. Fecchio , T. Peçanha , A. Andrade‐Lima , J. R. Halliwill , and C. L. M. Forjaz . 2018. “Postexercise Hypotension as a Clinical Tool: A “Single Brick” in the Wall.” Journal of the American Society of Hypertension 12, no. 12: e59–e64. 10.1016/J.JASH.2018.10.006. 30425018

[ejsc70006-bib-0004] Chobanian, A. V. , G. L. Bakris , H. R. Black , et al. 2003. “The Seventh Report of the Joint National Committee on Prevention, Detection, Evaluation, and Treatment of High Blood Pressure: The JNC 7 Report.” JAMA 289, no. 19: 2560–2572. 10.1001/JAMA.289.19.2560. 12748199

[ejsc70006-bib-0005] Christiani, M. , G. J. Grosicki , and A. A. Flatt . 2021. “Cardiac‐Autonomic and Hemodynamic Responses to a Hypertonic, Sugar‐Sweetened Sports Beverage in Physically Active Men.” Applied Physiology, Nutrition, and Metabolism = Physiologie Appliquee, Nutrition et Metabolisme 46, no. 10: 1189–1195. 10.1139/APNM-2021-0138. 33761293

[ejsc70006-bib-0006] Collier, S. R. , M. D. Diggle , K. S. Heffernan , E. E. Kelly , M. M. Tobin , and Bo Fernhall . 2010. “Changes in Arterial Distensibility and Flow‐Mediated Dilation After Acute Resistance vs. Aerobic Exercise.” Journal of Strength & Conditioning Research 24, no. 10: 2846–2852. 10.1519/JSC.0B013E3181E840E0. 20885204

[ejsc70006-bib-0007] De Souza, J. C. , R. A. Tibana , C. R. Cavaglieri , et al. 2013. “Resistance Exercise Leading to Failure versus Not to Failure: Effects on Cardiovascular Control.” BMC Cardiovascular Disorders 13, no. 1: 1–9. 10.1186/1471-2261-13-105/TABLES/3. 24252583 PMC3840620

[ejsc70006-bib-0008] de Tarso Veras Farinatti, P. , F. Y. Nakamura , and M. D. Polito . 2009. “Influence of Recovery Posture on Blood Pressure and Heart Rate After Resistance Exercises in Normotensive Subjects.” Journal of Strength & Conditioning Research 23, no. 9: 2487–2492. 10.1519/JSC.0B013E3181B25E48. 19910826

[ejsc70006-bib-0009] Esco, M. R. , and A. A. Flatt . 2014. “Ultra‐Short‐Term Heart Rate Variability Indexes at Rest and Post‐Exercise in Athletes: Evaluating the Agreement With Accepted Recommendations.” Journal of Sports Science and Medicine 13, no. 3: 535. https://pmc.ncbi.nlm.nih.gov/articles/PMC4126289/. 25177179 PMC4126289

[ejsc70006-bib-0010] Farinatti, P. , M. D. Polito , R. Massaferri , et al. 2021. “Postexercise Hypotension Due to Resistance Exercise Is Not Mediated by Autonomic Control: A Systematic Review and Meta‐Analysis.” Autonomic Neuroscience: Basic and Clinical 234: 102825. 10.1016/J.AUTNEU.2021.102825. 34118764

[ejsc70006-bib-0011] Figueiredo, T. , M. R. Rhea , M. Peterson , et al. 2015. “Influence of Number of Sets on Blood Pressure and Heart Rate Variability After a Strength Training Session.” Journal of Strength & Conditioning Research 29, no. 6: 1556–1563. 10.1519/JSC.0000000000000774. 25436620

[ejsc70006-bib-0012] Giles, D. , N. Draper , and W. Neil . 2016. “Validity of the Polar V800 Heart Rate Monitor to Measure RR Intervals at Rest.” European Journal of Applied Physiology 116, no. 3: 563–571. 10.1007/S00421-015-3303-9. 26708360 PMC4751190

[ejsc70006-bib-0013] Gomides, R. , R. Dias , D. Souza , et al. 2010. “Finger Blood Pressure During Leg Resistance Exercise.” International Journal of Sports Medicine 31, no. 8: 590–595. 10.1055/S-0030-1252054. 20432200

[ejsc70006-bib-0014] Güngör, A. K. , H. Topçu , M. I. Aldhahi , S. B. Al‐Mhanna , and M. Gülü . 2024. “Resistance Training to Muscle Failure With Variable Load Intensities: Implications for Post‐Exercise Blood Pressure and Heart Rate Variability in Trained Men.” Journal of Clinical Medicine 13, no. 8. 10.3390/JCM13082296. PMC11051069 38673569

[ejsc70006-bib-0015] Haff, G. , and N. Triplett . 2015. Essentials of Strength Training and Conditioning. 4th ed. Human Kinetics.

[ejsc70006-bib-0016] Haff, G. G. , R. T. Hobbs , E. E. Haff , W. A. Sands , K. C. Pierce , and M. H. Stone . 2008. “Cluster Training: A Novel Method for Introducing Training Program Variation.” Strength and Conditioning Journal 30, no. 1: 67–76. 10.1519/SSC.0B013E31816383E1.

[ejsc70006-bib-0017] Hedrick, A. 2019. Dumbbell Training. 2nd ed. Human Kinetics.

[ejsc70006-bib-0018] Holmes, C. J. , H. V. MacDonald , M. R. Esco , M. V. Fedewa , S. A. Wind , and L. J. Winchester . 2022. “Comparison of Heart Rate Variability Responses to Varying Resistance Exercise Volume‐Loads.” Research Quarterly for Exercise & Sport 93, no. 2: 391–400. 10.1080/02701367.2020.1851351. 33300852 PMC8768028

[ejsc70006-bib-0019] Hopkins, W. G. , S. W. Marshall , A. M. Batterham , and J. U. R. I. Hanin . 2009. “Progressive Statistics for Studies in Sports Medicine and Exercise Science.” Medicine & Science in Sports & Exercise 41, no. 1: 3–12. 10.1249/MSS.0B013E31818CB278. 19092709

[ejsc70006-bib-0020] Iglesias‐Soler, E. , D. A. Boullosa , E. Carballeira , et al. 2015. “Effect of Set Configuration on Hemodynamics and Cardiac Autonomic Modulation After High‐Intensity Squat Exercise.” Clinical Physiology and Functional Imaging 35, no. 4: 250–257. 10.1111/CPF.12158. 24774862

[ejsc70006-bib-0021] Koo, T. K. , and M. Y. Li . 2016. “A Guideline of Selecting and Reporting Intraclass Correlation Coefficients for Reliability Research.” Journal of Chiropractic Medicine 15, no. 2: 155–163. 10.1016/J.JCM.2016.02.012. 27330520 PMC4913118

[ejsc70006-bib-0022] Li, Y. , M. Bopp , F. Botta , et al. 2015. “Lower Body vs Upper Body Resistance Training and Arterial Stiffness in Young Men.” International Journal of Sports Medicine 36, no. 12: 960–967. 10.1055/S-0035-1549921/ID/R4573-0016/BIB. 26212244

[ejsc70006-bib-0023] Lovell, D. I. , R. Cuneo , and G. C. Gass . 2011. “The Blood Pressure Response of Older Men to Maximum and Sub‐Maximum Strength Testing.” Journal of Science and Medicine in Sport 14, no. 3: 254–258. 10.1016/J.JSAMS.2010.12.005. 21216668

[ejsc70006-bib-0024] Malik, M. , A., John Camm , J., Thomas Bigger , G., Breithardt , S., Cerutti , R. J., Cohen , et al. 1996. “Heart Rate Variability: Standards of Measurement, Physiological Interpretation, and Clinical Use.” Circulation 93, no. 5: 1043–1065. 10.1161/01.CIR.93.5.1043. 8598068

[ejsc70006-bib-0025] Mayo, X. , E. Iglesias‐Soler , E. Carballeira‐Fernández , and M. Fernández‐Del‐Olmo . 2016. “A Shorter Set Reduces the Loss of Cardiac Autonomic and Baroreflex Control After Resistance Exercise.” European Journal of Sport Science 16, no. 8: 996–1004. 10.1080/17461391.2015.1108367. 26568203

[ejsc70006-bib-0026] Mayo, X. , E. Iglesias‐Soler , J. Fariñas‐Rodríguez , M. Fernández‐del‐Olmo , and J. D. Kingsley . 2016. “Exercise Type Affects Cardiac Vagal Autonomic Recovery After a Resistance Training Session.” Journal of Strength & Conditioning Research 30, no. 9: 2565–2573. 10.1519/JSC.0000000000001347. 26817741

[ejsc70006-bib-0027] Okamoto, T. , M. Masuhara , and K. Ikuta . 2009. “Upper But Not Lower Limb Resistance Training Increases Arterial Stiffness in Humans.” European Journal of Applied Physiology 107, no. 2: 127–134. 10.1007/S00421-009-1110-X/TABLES/2. 19533164

[ejsc70006-bib-0028] Okuno, N. M. , R. E. Pedro , A. S. Leicht , S. de Paula Ramos , and F. Y. Nakamura . 2014. “Cardiac Autonomic Recovery After a Single Session of Resistance Exercise With and Without Vascular Occlusion.” Journal of Strength & Conditioning Research 28, no. 4: 1143–1150. 10.1519/JSC.0000000000000245. 24077384

[ejsc70006-bib-0029] Pareja‐Blanco, F. , D. Rodríguez‐Rosell , P. Aagaard , et al. 2020. “Time Course of Recovery From Resistance Exercise With Different Set Configurations.” Journal of Strength & Conditioning Research 34, no. 10: 2867–2876. 10.1519/JSC.0000000000002756. 30036284

[ejsc70006-bib-0030] Pickering, T. G. , J. E. Hall , L. J. Appel , et al. 2005. “Recommendations for Blood Pressure Measurement in Humans and Experimental Animals: Part 1: Blood Pressure Measurement in Humans: A Statement for Professionals From the Subcommittee of Professional and Public Education of the American Heart Association Council on High Blood Pressure Research.” Circulation 111, no. 5: 697–716. 10.1161/01.CIR.0000154900.76284.F6. 15699287

[ejsc70006-bib-0031] Polito, M. D. , and P. T. V. Farinatti . 2009. “The Effects of Muscle Mass and Number of Sets During Resistance Exercise on Postexercise Hypotension.” Journal of Strength & Conditioning Research 23, no. 8: 2351–2357. 10.1519/JSC.0B013E3181BB71AA. 19826288

[ejsc70006-bib-0032] Rúa‐Alonso, M. , X. Mayo , J. Mota , J. D. Kingsley , and E. Iglesias‐Soler . 2020. “A Short Set Configuration Attenuates the Cardiac Parasympathetic Withdrawal After a Whole‐Body Resistance Training Session.” European Journal of Applied Physiology 120, no. 8: 1905–1919. 10.1007/S00421-020-04424-3. 32583361

[ejsc70006-bib-0033] Rabbani, M. , H. Agha‐Alinejad , R. Gharakhanlou , A. Rabbani , and A. A. Flatt . 2021. “Monitoring Training in Women’s Volleyball: Supine or Seated Heart Rate Variability?” Physiology & Behavior 240: 113537. 10.1016/J.PHYSBEH.2021.113537. 34331956

[ejsc70006-bib-0034] Refalo, M. C. , E. R. Helms , D. L. Hamilton , and J. J. Fyfe . 2022. “Towards an Improved Understanding of Proximity‐To‐Failure in Resistance Training and its Influence on Skeletal Muscle Hypertrophy, Neuromuscular Fatigue, Muscle Damage, and Perceived Discomfort: A Scoping Review.” Journal of Sports Sciences 40, no. 12: 1369–1391. 10.1080/02640414.2022.2080165. 35658845

[ejsc70006-bib-0035] Rezk, C. C. , R. C. B. Marrache , T. Tinucci , D. Mion , and C. L. M. Forjaz . 2006. “Post‐Resistance Exercise Hypotension, Hemodynamics, and Heart Rate Variability: Influence of Exercise Intensity.” European Journal of Applied Physiology 98, no. 1: 105–112. 10.1007/S00421-006-0257-Y/TABLES/3. 16896732

[ejsc70006-bib-0036] Rúa‐Alonso, M. , X. Mayo , J. Rial‐Vázquez , J. Fariñas , A. Aracama , and E. Iglesias‐Soler . 2022. “Hemodynamic Response During Different Set Configurations of a Moderate‐Load Resistance Exercise.” International Journal of Sports Medicine 43, no. 13: 1118–1128. 10.1055/A-1843-8778. 35508201

[ejsc70006-bib-0037] Sale, D. G. , D. E. Moroz , R. S. McKelvie , J. D. MacDougall , and N. McCartney . 1994. “Effect of Training on the Blood Pressure Response to Weight Lifting.” Canadian Journal of Applied Physiology 19, no. 1: 60–74. 10.1139/H94-004. 8186763

[ejsc70006-bib-0038] Selig, S. , M. Carey , D. Menzies , et al. 2004. “Moderate‐Intensity Resistance Exercise Training in Patients With Chronic Heart Failure Improves Strength, Endurance, Heart Rate Variability, and Forearm Blood Flow.” Journal of Cardiac Failure 10, no. 1: 21–30. 10.1016/S1071-9164(03)00583-9. 14966771

[ejsc70006-bib-0039] Stanley, J. , J. M. Peake , and M. Buchheit . 2013. “Cardiac Parasympathetic Reactivation Following Exercise: Implications for Training Prescription.” Sports Medicine 43, no. 12: 1259–1277. 10.1007/S40279-013-0083-4. 23912805

[ejsc70006-bib-0040] Tarvainen, M. P. , P. O. Ranta‐aho , and P. A. Karjalainen . 2002. “An Advanced Detrending Method With Application to HRV Analysis.” IEEE Transactions on Biomedical Engineering 49, no. 2: 172–175. 10.1109/10.979357. 12066885

[ejsc70006-bib-0041] Toner, M. M. , E. L. Glickman , and W. D. McARDLE . 1990. “Cardiovascular Adjustments to Exercise Distributed Between the Upper and Lower Body.” Medicine & Science in Sports & Exercise 22, no. 6: 773–778. 10.1249/00005768-199012000-00007. 2287254

[ejsc70006-bib-0042] Tufano, J. J. , L. E. Brown , and G. G. Haff . 2017. “Theoretical and Practical Aspects of Different Cluster Set Structures: A Systematic Review.” Journal of Strength & Conditioning Research 31, no. 3: 848–867. 10.1519/JSC.0000000000001581. 27465625

[ejsc70006-bib-0043] Tumminello, N. 2022. Strength Zone Training: The Most Effective Method for Maximizing Muscle Development. Human Kinetics.

[ejsc70006-bib-0044] Von Klot, S. , M. A. Mittleman , D. W. Dockery , et al. 2008. “Intensity of Physical Exertion and Triggering of Myocardial Infarction: A Case‐Crossover Study.” European Heart Journal 29, no. 15: 1881–1888. 10.1093/EURHEARTJ/EHN235. 18534976

[ejsc70006-bib-0045] Whelton, P. K. , R. M. Carey , W. S. Aronow , et al. 2018. “2017 ACC/AHA/AAPA/ABC/ACPM/AGS/APhA/ASH/ASPC/NMA/PCNA Guideline for the Prevention, Detection, Evaluation, and Management of High Blood Pressure in Adults: A Report of the American College of Cardiology/American Heart Association Task Force on Clinical Practice Guidelines.” Journal of the American College of Cardiology 71, no. 19: e127–e248. 10.1016/J.JACC.2017.11.006. 29146535

